# Rigorous and thorough bioinformatic analyses of olfactory receptor promoters confirm enrichment of O/E and homeodomain binding sites but reveal no new common motifs

**DOI:** 10.1186/1471-2164-12-561

**Published:** 2011-11-15

**Authors:** Janet M Young, Ralf M Luche, Barbara J Trask

**Affiliations:** 1 Division of Human Biology, Fred Hutchinson Cancer Research Center, Seattle, WA 98109, USA; 2 Present address: Division of Clinical Research, Fred Hutchinson Cancer Research Center, Seattle, WA 98109, USA

**Keywords:** Olfactory receptor, comparative genomics, transcriptional regulation, transcription factor binding site, position weight matrix; homeodomain

## Abstract

**Background:**

Mammalian olfactory receptors (ORs) are subject to a remarkable but poorly understood regime of transcriptional regulation, whereby individual olfactory neurons each express only one allele of a single member of the large OR gene family.

**Results:**

We performed a rigorous search for enriched sequence motifs in the largest dataset of OR promoter regions analyzed to date. We combined measures of cross-species conservation with databases of known transcription factor binding sites and *ab initio *motif-finding algorithms. We found strong enrichment of binding sites for the O/E family of transcription factors and for homeodomain factors, both already known to be involved in the transcriptional control of ORs, but did not identify any novel enriched sequences. We also found that TATA-boxes are present in at least a subset of OR promoters.

**Conclusions:**

Our rigorous approach provides a template for the analysis of the regulation of large gene families and demonstrates some of the difficulties and pitfalls of such analyses. Although currently available bioinformatics methods cannot detect all transcriptional regulatory elements, our thorough analysis of OR promoters shows that in the case of this gene family, experimental approaches have probably already identified all the binding factors common to large fractions of OR promoters.

## Background

Mammals can detect and discriminate many thousands of odorous molecules in our environments using millions of neurons in the olfactory epithelium. These neurons express members of one of the largest gene families in the mammalian genome, the olfactory receptors (ORs) [1]. In mouse, this gene family comprises ~1150 apparently functional genes and ~300 pseudogenes [2,3] (Additional File 1). As a result of the gene family's ongoing expansion by tandem duplication, most mouse ORs are found in the genome in clusters of up to ~300 genes with ORFs spaced ~21 kb apart on average [2]. Although some clear one-to-one orthologs can be identified across species, extensive post-speciation duplication has resulted in many ORs having one-to-many or many-to-many orthologous relationships. Phylogenetic trees show that ORs can be divided into two major classes, class I and class II ORs [4] (with ~130 and ~1020 intact ORs in mouse, respectively). While the ligands recognized by most mouse ORs remain unknown, available odorant-response profiles suggest that class I ORs recognize odorants that are more hydrophilic than those recognized by class II [5].

Each neuron in the olfactory epithelium expresses only one member of the OR gene family; furthermore, only one of the two parental alleles of the selected OR is expressed in each cell [6-8]. Individual OR genes are therefore expressed in only a small subset of the neurons of the olfactory epithelium. This "singular" mode of expression ensures that different neurons respond to different odorants and that a distinct pattern of neuronal activation is generated for each odorant, thereby allowing for perceptual discrimination. Neurons expressing a given OR tend to be confined to one of five regions of the olfactory epithelium (four originally described "zones" and the "OR37 patch") [9-11], although recent studies suggest that these zones are less clearly defined than previously thought and can partially overlap [12]. Each cell's choice among the set of "zone-appropriate" ORs appears to be stochastic [6,9]. However, some ORs are expressed in many more cells and/or at higher per-cell levels than others [8,9,13], implying that some ORs may have "stronger" promoters than others.

Transgene studies have shown that a small region of a few hundred base pairs upstream of the OR transcription start site (TSS) can act as a minimal promoter to drive transcription of a reporter construct in a pattern that seems mostly faithful to that of the endogenous gene, although occasional differences are observed in zonal restriction of transgenes [14-16]. It therefore seems reasonable to expect that regions close to the TSS will contain functional sequence motifs involved in transcriptional activation of OR genes. One might expect the existence of transcription factors common to all ORs and/or transcription factors specific for different subsets of ORs, perhaps those expressed in certain zones of the olfactory epithelium, or certain phylogenetic subfamilies such as class I or class II ORs.

Two classes of transcription factors have been identified that appear important in OR activation: the O/E family and homeodomain proteins. Binding sites for O/E and homeodomain proteins have been identified in many OR promoter regions, both experimentally and using bioinformatic methods [14,16-21]. The O/E family comprises four helix-loop-helix (HLH) transcription factors that are expressed throughout the olfactory epithelium: *O/E-1 *(also known as *Olf-1*, *Ebf1*), *O/E-2*, *O/E-3 *and *O/E-4 *[22,23]. The four family members appear to have similar DNA recognition specificities and may form heterodimers with one another. The pattern of expression in non-olfactorytissues differs between the four O/E family members, as does their strength of transcriptional activation [22,23]. Mice lacking *O/E-1*, *O/E-2 *or *O/E-3 *die prematurely; their olfactory epithelia appear normal on a gross level, but the projection of olfactory neurons to the brain appears disrupted, perhaps implying subtle shifts in the expression pattern of O/E target genes, including ORs [24,25]. The O/E family members exhibit at least partial redundancy with one another [22]. A zinc-finger protein, Roaz, might interact with O/E family members, perhaps preventing them from acting as transcriptional activators [26].

Two homeodomain proteins are known to be involved in OR regulation: Lhx2 and Emx2. The LIM-homeodomain protein Lhx2 was first identified as an olfactory transcription factor by virtue of its binding to the promoter region of one OR, *M71 *[27]. Studies of mice mutant for *Lhx2 *show that it is important for transcription of all class II ORs but not most class I ORs [28,29]. Notably, the two class I ORs that were tested whose expression is affected in *Lhx2 *knockout mice are normally expressed in a dorsal region of the olfactory epithelium along with class II ORs, whereas the unaffected class I genes are normally expressed in a more ventral region [28]. *Lhx2 *itself is expressed throughout the olfactory epithelium, most strongly in neuronal precursor cells [27]. A second homeodomain protein, Emx2, has also been implicated in OR transcriptional control. It also binds the *M71 *promoter and is expressed throughout the olfactory epithelium [27]. Most OR genes show dramatically reduced transcript levels in *Emx2 *knockout mice [30]. A third homeodomain protein, Dlx5, is important for proper olfactory neuron axon targeting [31]; one possible explanation is that it is involved in OR regulation, although OR expression has not been examined in *Dlx5 *mutant mice.

Although candidate activation signals common to most or all ORs have been identified, the singular mode of expression has not yet been fully explained despite extensive efforts. An enhancer-like element, the "H region" is required for transcription of its neighboring ORs and might act as a locus control region (LCR) to select a single gene for expression from the nearby OR cluster [32]. The H region also shows nuclear co-localization with promoters of actively transcribed ORs on other chromosomes [33], leading to the hypothesis that the H region could also act as an enhancer/LCR in *trans *for ORs throughout the genome [33]. However, experiments in mice in which the H region is deleted show that, while it is essential for transcription of nearby ORs, it appears dispensable for the transcription of more distant ORs and ORs on other chromosomes [34,35]. A negative-feedback mechanism might partially explain the singular mode of expression; once an OR is chosen for expression (perhaps stochastically), negative feedback seems to operate to prevent transcription from other OR loci, a mechanism that might involve the OR protein-coding region to send and/or receive repressive [32,36-38]. Another attractive hypothesis that had been proposed to explain singular expression is somatic recombination into an active locus, analogous to the role of recombination in generating lymphocyte diversity and singular expression of T-cell receptors and immunoglobulins. However, elegant nuclear transfer experiments show that recombination is very unlikely to play a role in OR gene choice [39,40]. Recently published work has shown that epigenetic marks may play a key role in the mechanism of OR silencing and expression [41].

The goal of the present study is to perform rigorous statistical and bioinformatics analyses on OR promoter regions in an attempt to identify additional common DNA sequence motifs that might function in positive or negative transcriptional control of the gene family. Experimental studies of OR regulation are inherently difficult, because cells expressing each OR are rare (~1/1000 neurons) and dispersed throughout a relatively small tissue. Bioinformatic studies could therefore be a useful complementary tool. A previous study [20] used *ab initio *motif-identification tools on 198 mouse OR promoters to show that motifs similar to O/E and homeodomain sites are enriched in OR promoters. Our study greatly extends this earlier work, adding large-scale examination of databases of known transcription factor binding sites and the powerful tool of comparative genomics, as well as using a larger dataset of more than 300 OR promoter sequences.

The identification of functionally important sequences in a set of promoter regions of interest using bioinformatic methods is a difficult task, largely due to issues of poor signal-to-noise ratio. Transcription factors tend to recognize short and often degenerate sequences, making it difficult to identify over-represented candidate binding sites using so-called *ab initio *motif-finding methods. This degeneracy also makes it difficult to scan a genomic sequence using known motifs and computationally distinguish true binding sites from false positives. An increasing number of studies (e.g., [42,43]) have harnessed the power of comparative genomics to help sift signal from noise. Sequences that are functionally important are often conserved during evolution, acquiring mutations at a slower rate than non-functional sequences, and can thus be recognized in multi-species alignments as regions with fewer sequence changes than expected given the phylogenetic relationship between the species studied [44,45]. Until now, comparative sequence analysis has not been applied in a systematic way to a large collection of OR promoters.

Together, the approaches we describe here provide a rigorous and thorough examination of enriched and conserved sequences in promoters of the OR gene family. We found strong evidence supporting the importance of the O/E family of transcription factors, homeodomain factors and TATA boxes but found no additional enriched common sequence motifs, indicating that experimental approaches are more likely to yield the next breakthrough in the field of OR transcriptional regulation.

## Results

### General characteristics of OR promoter regions

In order to detect signals in nucleotide sequences that might be important in controlling OR transcription, we needed a dataset of well-defined OR transcription start sites (TSSs). ORs typically have intronless protein-coding regions, but their 5' untranslated regions (UTRs) usually contain introns [1,13]. The genomic locations of the 5' exons, introns and TSSs cannot easily be predicted computationally nor derived from high-throughput EST or CAGE experiments, which have overlooked OR transcripts due to issues of tissue availability and transcript abundance. Targeted approaches have therefore been necessary to yield locations of upstream exons, TSSs and promoters for the OR genes and have been performed almost exclusively using mouse tissues.

Michaloski *et al. *performed 5'-RACE for 198 mouse ORs; sequences obtained from these RACE products can be mapped to the mouse genome assembly to yield putative locations for the corresponding TSSs and promoters [20]. We also collected 5'-RACE data from various other studies of smaller numbers of ORs (Additional File 2). In addition, we previously performed a hybridization-based cDNA library screen to identify putative TSSs for more than 300 ORs [13]. A potential concern of using cDNA-defined TSSs is that some cDNAs in the library may not be full-length. Examination of the 90 ORs for which both RACE-defined and cDNA-defined TSSs are available shows that EST completeness is not a major concern; in 57/90 (63%) cases the EST- and RACE-defined TSSs map within 50 bp of one another. In the remaining 33 cases the RACE sequence(s) RACE data imply a TSS further upstream than the EST sequence in 21 of those 33 ORs, but for the other 12, EST sequences extend further upstream. For an OR gene where different methods or cDNA clones imply different TSS locations, it is quite possible that several alternate start sites truly exist, as has been observed for many genes [46]; however, it is also possible that some of the sequenced clones are not full-length. We have therefore taken the conservative approach of analyzing only the most upstream TSS predicted for each gene. Combining all the data sources mentioned above, we can define putative TSS locations for 432 intact ORs (see Methods), which represents more than one-third of the 1151 intact OR genes we find in the July 2007 version of the mouse genome assembly (Additional File 1). Only 26 of these 432 genes are class I ORs (partly due to bias of the degenerate primers used in both large-scale studies towards class II ORs [13,20]), whereas 406 are class II ORs. This primer bias means that the statistical tests we describe below will be more powerful for class II ORs than for class I ORs and make comparisons between those two groups difficult.

Some OR genes are the products of relatively recent genomic duplications involving a stretch of genomic DNA that can include the promoter region [47]. It is important for the statistical validity of many of the analyses we describe below to include only one member of any group of recent gene/promoter duplicates, so that (for example) a transcription factor binding site observed in similar locations in multiple promoter sequences is likely to be regionally over-represented due to functional considerations, rather than because those promoters share much of their sequence due to recent duplication (i.e., we wanted to ensure independence in our statistical tests). Therefore, we applied an additional filtering step to the promoter dataset to ensure that it contained no pairs of recently duplicated ORs (see Methods). After such filtering, our promoter dataset contained 314 putative OR TSSs, comprising 24 class I ORs and 290 class II ORs. Although these TSS locations might include a minority that are defined imprecisely or perhaps even wrongly, in order to keep the following text as clear as possible, we have omitted the words "putative", "candidate", etc., each time we refer to the TSSs we have analyzed. We performed our analyses on two alternative datasets of regions: (a) the 200-bp region immediately preceding these TSSs, because many of the functional elements important for transcription should be found close to the TSS [14,16] and motif searches should be more powerful with smaller sequence sets; and (b) a larger 500-bp region immediately preceding the TSSs, because it is possible that the biologically active promoter region might be larger than 200bp for some fraction of the ORs. We refer to these 200-bp or 500-bp regions below as "promoters" for simplicity, although this is surely a very rough approximation to the true promoter regions.

**Figure 1:**
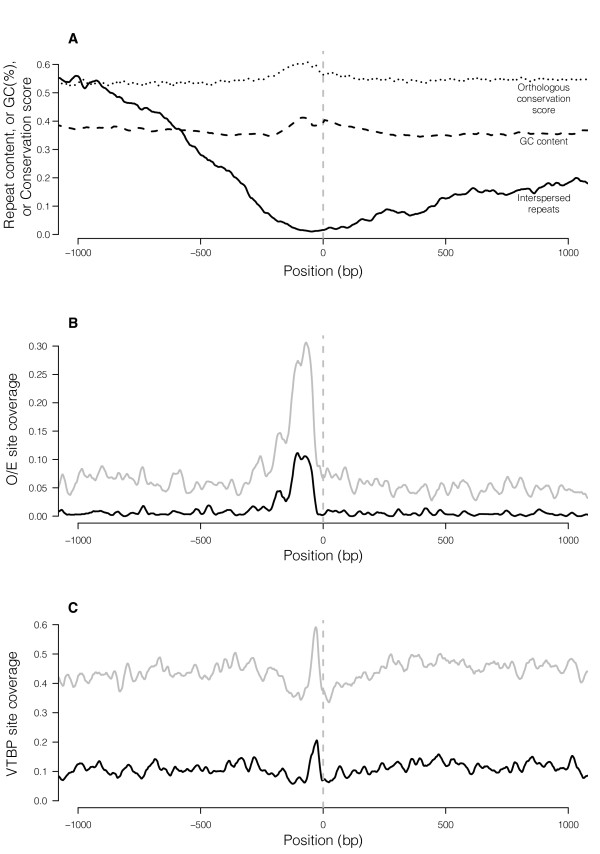
**General characteristics of 314 putative OR promoter regions**. Panel A. Interspersed repeat content, GC content and orthologous conservation scores in a 2-kb region surrounding the putative TSSs of 314 ORs. GC content is calculated in 50-bp windows across each sequence (with a 10-bp slide), then averaged across all sequences in the dataset, plotting values at the center of each window. Interspersed repeat content was determined by RepeatMasker and the proportion of promoters containing a repeat element at each base position relative to the TSS was calculated (averaged over 20-bp windows, sliding along promoters 1 bp at a time). An orthologous conservation score was calculated using SCONE [45]: the value plotted is 1 - P-value (SCONE output, see Methods) and is averaged over 20-bp windows, sliding along promoters 1 bp at a time. The vertical dashed gray line represents the predicted TSS. Panel B: Distribution of predicted O/E binding sites using MatInspector's default parameters ("opt", black line) or a reduced stringency ("opt-0.1", gray line) in the 314 sequences. Coverage is calculated as the proportion of promoter sequences containing a predicted O/E binding site at each base-pair, averaged over 20-bp windows, sliding along promoters 1 bp at a time. Panel C: Distribution of predicted TATA boxes using MatInspector's optimized score threshold (black line) or a less stringent threshold (gray line) (20-bp windows, 1-bp slide).

These 314 TSSs are 4.2 kb away from their corresponding translational start sites on average (range 200 bp - 24 kb, standard deviation 3.2 kb). Figure 1A shows that repeat content drops dramatically approaching the predicted TSSs; fewer than 2% of bases in the last 200 bp before these TSSs are recognized by RepeatMasker as repetitive elements. In contrast, cross-species conservation scores rise approaching the TSS (Figure 1A). These patterns of repeat content and conservation scores indicate that the majority of the candidate TSSs are indeed likely to be real. Considering the 4 kb centered at the TSS, the GC content of the OR promoter regions averages 36.4%, much lower than the genome-wide average for mouse of ~42% [48]. The rise and dip in GC content very close to the TSS (Figure 1A) is partly, but not entirely, due to the high frequency of GC-rich O/E sites and AT-rich TATA boxes in those regions (see below, Figure 1B, 1C). No predicted CpG islands lie within 2 kb of any of these 314 TSSs. If we analyze class I and class II ORs separately, these patterns of GC content, repeat content, conservation score and O/E and TATA-box enrichment hold for both classes (data not shown); unfortunately not enough TSSs are defined for class I genes to rigorously test for differences in the enrichment levels between classes.

### O/E sites and TATA boxes are enriched in OR promoters

We scanned the promoter sequences using a collection of position weight matrices (PWMs) representing known transcription factor binding sites. Several such databases and search algorithms exist; we chose to use the MatBase database and the corresponding MatInspector search program [49] (Genomatix Software GmbH, Munich, Germany). As discussed above, most transcription factor binding sites recognize short, degenerate sequence motifs, and methods for scanning genomic sequences for PWM matches suffer from signal-to-noise problems. Most search algorithms score every position of a genomic sequence for its similarity to the PWM, and then take matches exceeding a certain score threshold as candidate transcription factor binding sites. MatInspector's default parameters use an optimized score threshold for each PWM that is designed to minimize false positives (calibrated by minimizing the number of matches in non-promoter regions) in order to maximize the proportion of predicted sites that represent true positive calls. Although this approach is appropriate in some studies (e.g., prior to time-consuming experimental tests of individual site predictions), minimizing false positives inevitably also results in a high false-negative rate. In other situations, it might be more important to minimize false negatives, such that a less stringent threshold might be appropriate. Our study is one such situation: the veracity of individual sites is less important, because statistical methods will be used to select motifs that are enriched across multiple promoters. We therefore scanned the promoter sequences using MatInspector's less stringent score threshold ("opt-0.1", see Methods) as well as its optimized score threshold (referred to below as "opt"). For some matrices, like those representing O/E (Figure 1B), the reduced stringency results appear useful, as many more sites are detected close to the TSS without a high level of background noise further from the promoter sequence. However, for other matrices, like those representing the TATA-box, the background level is prohibitively high when the score threshold is reduced (Figure 1C).

The MatBase database groups related PWMs into families to simplify interpretation of results (see Methods). For every PWM family, we plotted the spatial distribution of predicted binding sets near TSSs in our dataset. We first examined the distribution of matches to MatBase's V$NOLF family, which comprises two similar matrices representing binding sites for the O/E family of transcription factors [50], both of which are fairly long (21 and 18 bp) and quite specific. As expected [20], we find a clear peak of enrichment of O/E sites close to the predicted TSSs (Figure 1B). Cross-species conservation analysis indicates that most O/E sites predicted even using MatInspector's less stringent parameters are conserved (Additional File 3). Thus, for this matrix family, the opt-0.1 predictions yield a better true-positive rate than the default opt predictions and still do not suffer from a high false-positive rate. In order to estimate the number of real O/E sites, we used cross-species conservation to filter MatInspector's opt-0.1 O/E predictions (Additional File 3, Methods). We find that 246 of the 314 200-bp promoters examined (78%) contain a conserved O/E site in the 200 bp closest to the TSS; many promoters contain more than one such site (range 0-4 O/E sites per 200-bp promoter; mean 1.38 sites).

We also noticed a clear peak of enrichment of O$VTBP-family matrix matches very close to the predicted transcription start sites (Figure 1C). O$VTBP matrices represent the vertebrate TATA box and are short and quite degenerate. This degeneracy results in a large number of background matches, making the number of truly functional sites difficult to estimate. Considering just the -60 to -10 region (the region represented by this peak of enrichment), MatInspector calls an "opt" O$VTBP site for 39% of the promoters in our set; however, there is a non-trivial rate of finding these sites in other parts of the promoter region. Although we cannot determine what proportion of OR promoters contain a functional TATA-box, such sequences are clearly enriched near OR TSSs. Previous studies have classified OR promoters as being mostly TATA-less [20,51,52], but the enrichment we observe indicates that TATA boxes are likely used in at least a subset of OR promoters.

### Statistical tests reveal a number of transcription factors whose binding sites are enriched in OR promoters

**Figure 2:**
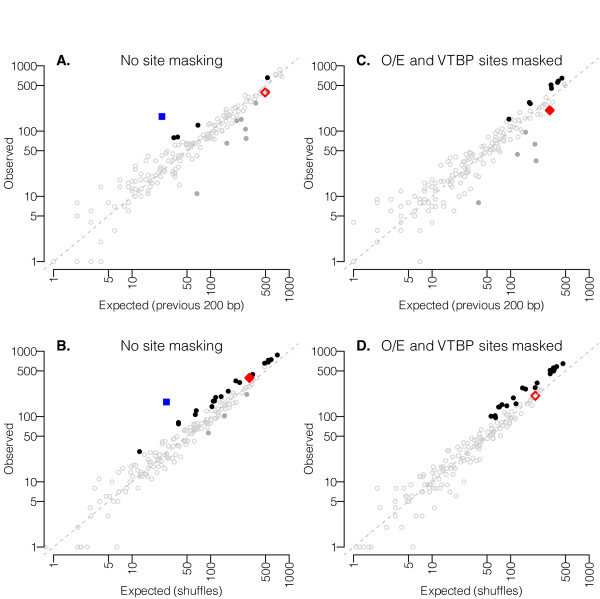
**Number of predicted TF binding sites within 200-bp of the TSS compared to background sequences**. Each log-scale plot shows the total number of predicted binding sites in the set of 314 200-bp promoter regions on the y-axis. The x-axis represents the number of sites found in the preceding 200-bp regions (upper panels, A and C) or the average number of sites predicted in 10,000 shuffled sequence datasets (lower panels, B and D). Each data point represents a family of transcription factor matrices in the MatBase database; MatInspector analysis was performed using the default parameters (see Additional File 4 for analysis using less stringent parameters, and Table 1 and Additional Files 5 and 6 for full results and matrix family names). Panels A and B (left) show results before masking O/E sites and TATA boxes, and panels C and D (right) show analysis after masking those sites. Data points for which one of the two values is 0 are not plotted (none shows statistically significant enrichment). Solid black symbols represent matrix families showing statistically significant enrichment; solid gray symbols represent matrix families showing statistically significant depletion; the blue square symbol highlights the V$NOLF family of matrices representing the O/E binding site (as expected, no O/E sites were predicted after applying the mask, so O/E does not appear in panels C and D); the red diamond symbol highlights the O$VTBP family of matrices representing TATA boxes.

We examined similar plots of spatial distributions for predicted binding sites of all other PWM families in the MatBase database. Some show enrichment close to the TSS, but to a lower level than was observed for O/E. In order to assess the statistical significance of enrichment for each matrix family, we performed two statistical tests comparing the number of predicted sites observed in the 200 bp preceding the TSS with the number of sites found in two types of "negative-control" sequences (Figure 2, Additional File 4; Methods). Our first test looks for enrichment compared to a nearby region; we used a binomial test to compare the number of predicted sites in the 200 bp upstream of the TSS with the number in the preceding 200-bp region (i.e., -200 bp to -400 bp relative to the TSS). For example, for V$NOLF at the "opt-0.1" stringency, we find 653 matches in the 200 bp preceding the TSSs of the 314 non-redundant ORs, but only 198 matches in the preceding 200 bp, representing ~3.3-fold enrichment. A one-tailed binomial test tells us that the likelihood of observing such extreme skew by chance is < 10^-57 ^(Table 1).

**Table 1:** Summary of statistical tests for 200-bp putative promoter region

					Comparison to previous 200bp region			Comparison to shuffled sequences			Conservation tests		
MatBase matrix family	MatBase family description	Consensus of arbitrarily chosen PWM	Number of predicted sites in 200bp promoter region	Number of 200bp promoters containing > = 1 site	Number of sites expected	Enrichment compared to expected number	Enrichment p-value	Number of sites expected	Enrichment compared to expected number	Enrichment p-value	p-value: whether whole sites are more conserved than local backgrounc	p-vaiue: whether core nucleotides are more conserved than local background	Significance levels of three tests
Before masking O/E and VTBP sites, default Matlnspector parameters, selected matrices													
V$NOLF	Neuron-specific-olfactory factor	nncdabTCCCyngrgarbnkgn	167	137	24	6.96	7.4E-28	27.4	6.1	<0.0001	5.2E-22	2.6E-54	X X X
O$VTBP	Vertebrate TATA binding protein factor	staTAAAwrnn	391	211	494	0.79	1	311	1.26	<0.0001	1	0.65	. X .
V$IKRS	Ikaros zinc finger family	yyTGGGagr	123	104	69	1.78	5.9E-5	65.6	1.88	<0.0001	2.2E-4	4.9E-14	X X X

Before masking O/E and VTBP sites, less stringent Matlnspector parameters (opt-0.10), selected matrices													
V$NOLF	Neuron-specific-olfactory factor	nncdabTCCCyngrgarbnkgn	653	265	198	3.3	9.6E-58	239.6	2.73	<0.0001	2.7E-64	3.4E-130	X X X
O$VTBP	Vertebrate TATA binding protein factor	staTAAAwrnn	2388	309	2972	0.8	1	2119.1	1.13	<0.0001	1	0.99	. X .
V$IKRS	Ikaros zinc finger family	yyTGGGagr	963	297	622	1.55	5E-18	710.3	1.36	<0.0001	1.6E-29	1.7E-73	X X X

After masking O/E and VTBP sites, default Matlnspector parameters, matrices significant in all three tests													
V$ARID	AT rich interactive domain factor	AATAccvm	140	94	89	1.57	4.6E-4	71.9	1.95	<0.0001	0.53	0.0021	+ X +
V$ATBF	AT-binding TF	hhwkrttantAATTahh	101	69	68	1.49	0.0068	56.3	1.8	<0.0001	0.069	7.4E-08	+ X X
V$BCDF	Bicoid-like homeodomain TFs	abnyTAATcmnv	152	119	102	1.49	0.001	131.5	1.16	0.0401	4.7E-17	4.1E-20	+ + X
V$BRN5	Brn-5 POU domain factors	gCATAawttat	327	165	282	1.16	0.037	217.5	1.5	<0.0001	0.015	5.3E-09	+ X X
V$CART	Cart-1 cartilage homeoprotein 1	cTAATtrnsynattan	452	183	331	1.37	8.7E-6	318.7	1.42	<0.0001	2.7E-17	2.1E-30	X X X
V$DLXF	Distal-less homeodomain TFs	nntAATTan	274	129	173	1.58	1.0E-6	141.8	1.93	<0.0001	1.9E-20	2.7E-35	X X X
V$HBOX	Homeobox TFs	raaTTTAattgaa	510	192	327	1.56	1.3E-10	317.9	1.6	<0.0001	4.3E-17	2.7E-30	X X X
V$HOMF	Homeodomain TFs	mCTAAttnn	646	214	449	1.44	1.4E-09	463.2	1.39	<0.0001	8.6E-4	1.4E-13	X X X
V$HOXF	Paralog hox genes 1-8, clusters A, B, C, D	nnamTAATgrggrwnn	583	204	404	1.44	6.7E-09	385.2	1.51	<0.0001	2.3E-09	9.3E-26	X X X
V$LHXF	Lim homeodomain factors	nntwwttAATTaatnn	557	187	396	1.41	1.0E-7	350.4	1.59	<0.0001	1.8E-08	2.8E-28	X X X
V$MYOD	Myoblast determining factors	mrgCARCwgswg	30	20	13	2.31	0.0069	16.3	1.84	0.0042	0.017	0.51	+ + +
V$NKX1	NK1 homeobox TFs	wgnrcyAATTrgygsnn	140	75	89	1.57	4.6E-4	70.2	1.99	<0.0001	1.2E-13	9.9E-21	+ X X
V$NKX6	NK6 homeobox TFs	TTAAttac	263	151	178	1.48	3.0E-5	155.1	1.7	<0.0001	1.3E-07	6.1E-13	X X X
V$PAXH	PAX homeodomain binding sites	aawaATTAnn	152	68	95	1.6	1.7E-4	77.5	1.96	<0.0001	0.0015	2.5E-10	X X X
V$PDX1	Pancreatic and intestinal homeodomain TF	rnTAATtagync	193	96	131	1.47	3.4E-4	108.4	1.78	<0.0001	1.4E-6	8.2E-16	+ X X

After masking O/E and VTBP sites, less stringent Matlnspector parameters (opt-0.10), matrices significant in all three tests													
V$AP4R	AP4and related proteins	wgaryCAGCtgyggnc	121	74	61	1.98	5.1E-6	99	1.22	0.0321	7.7E-08	0.061	X + X
V$DICE	Downstream Immunoglobulin Control Element	kgtySTCTccacag	186	134	135	1.38	0.0026	138.1	1.35	<0.0001	0.0026	0.2	+ X +
V$DLXF	Distal-less homeodomain TFs	nntAATTan	1252	274	1149	1.09	0.019	1041.7	1.2	<0.0001	0.0004	1.3E-18	+ X X
V$HAND	Twist subfamily of class B bHLH TFs	ccagaTGGCcccccn	696	252	537	1.3	3.3E-6	619.8	1.12	0.0048	0.0067	0.0019	X + +
V$NKX1	NK1 homeobox TFs	wgnrcyAATTrgygsnn	783	236	619	1.26	6.6E-6	634.4	1.23	<0.0001	9.9E-12	1E-21	X X X
V$PAX5	PAX-5 B-cell-specific activator protein	bcnnnrNKCAnbgnwgnrkrgc	227	139	180	1.26	0.011	192.2	1.18	0.0085	0.09	0.025	+ + +
V$PAX6	PAX-4/PAX-6 paired domain binding sites	GCASbswtgmgtgmn	664	249	555	1.2	9.8E-4	617	1.08	0.0354	0.011	0.0022	+ + +
V$PAXH	PAX homeodomain binding sites	aawaATTAnn	999	247	889	1.12	0.0061	767.1	1.3	<0.0001	4.5E-09	2E-20	+ X X
V$PDX1	Pancreatic and intestinal homeodomain TF	rnTAATtagync	1013	257	828	1.22	8.9E-6	743.9	1.36	<0.0001	2.6E-5	3.7E-25	X X X
V$PTF1	Pancreas TF 1, heterotrimeric TF	bmcaCCTGyvktkttycccrw	125	95	93	1.34	0.018	100.9	1.24	0.0102	0.015	0.17	+ + +
V$SIX3	Sine oculis homeobox homolog 3	nnrhnknTAATswcwncnstv	647	254	574	1.13	0.02	515.5	1.26	<0.0001	1.8E-07	6.6E-28	+ X X

Results of our statistical tests for enrichment and conservation are provided for selected matrix families before and after masking O/E sites and TATA boxes. Before masking, we provide results only for selected matrix families that we discuss in the text. After masking, we provide results for any matrix family that appeared statistically significant in all three tests before applying the Bonferroni correction for multiple testing. Additional Files 5, 6, 10 and 11 give results for all matrix families, as well as for all individual matrices. Additional Files 7, 8, 9, 12 and 13 give results of analogous tests for 500-bp putative promoter regions. P-values provided here are not corrected for multiple testing, but in selecting matrices for further discussion we used the conservative Bonferroni correction. For each matrix family, we provide the description from MatBase, using the abbreviation TF for transcription factor. We also provide the consensus sequence (using IUPAC degeneracy codes) of an arbitrarily chosen matrix from each family.

The "significance level" column summarizes the results of the three statistical tests in the following order: (a) enrichment versus previous 200-bp region (b) enrichment versus shuffled sequences (c) conservation scores versus surrounding nucleotides, counting whichever of the sites test or the cores test proved more significant (see Methods). The "." symbol indicates not significant; the "+" symbol indicates significance level of p < = 0.05 before applying Bonferroni correction; and the "X" symbol indicates that the p-value remains significant after applying the Bonferroni correction.

In the second test, we shuffled each 200-bp promoter sequence, maintaining its mono- and di-nucleotide composition (Methods). We performed MatInspector scans on the 314 shuffled sequences and compared the number of matches found for each PWM family in the true promoters with the number found in their shuffled counterparts. We repeated this process with 10,000 datasets of shuffled sequences. The proportion of shuffled sets in which we saw an equal or higher number of predicted transcription factor (TF) binding sites as seen in the real data provides an estimate of how likely the observed number of matrix matches would be seen by chance in sequence of similar nucleotide composition. Comparison of the number of predicted sites in real sequences with the mean number of predicted sites in the shuffled sets gives an estimate of the enrichment level. For example, there are 653 matches to V$NOLF family matrices ("opt-0.1") in the 200 bp upstream of the TSS in the real promoter set; in 10,000 shuffled versions of the same dataset, there are on average 239.6 V$NOLF sites (range 184-312). This method therefore estimates ~2.7-fold enrichment (Table 1). Because none of the 10,000 shuffled datasets contained as many predicted V$NOLF sites as the real promoters, we estimate the chance of seeing 167 matches by chance as being < 1/10,000 (< 0.0001) (using a conservative Bonferroni correction, our threshold for choosing significant motifs is p = 0.000282).

Although the O/E motif is significantly enriched according to both tests, the observed TATA-box enrichment (Figure 1C) is significant only when compared to shuffled sequences (p < 0.0001) and not by the binomial test comparing the 200-bp promoters to the preceding 200-bp regions (Table 1). In fact, there are fewer sites in the 200 bp upstream of the TSSs (391 sites) than in the preceding 200 bp (494 sites). This observation illustrates two limitations of our tests: (a) comparing a region as large as 200 bp may be insensitive when true enrichment peaks are more narrowly localized, and (b) the fluctuation in GC-content approaching the OR-TSSs can affect background levels of some motifs, especially short, degenerate AT-rich (or GC-rich) motifs. In the TATA-box case, enrichment at ~-60bp to ~-10bp is balanced by slight depletion in other parts of the 200-bp region due to raised GC-content.

**Figure 3:**
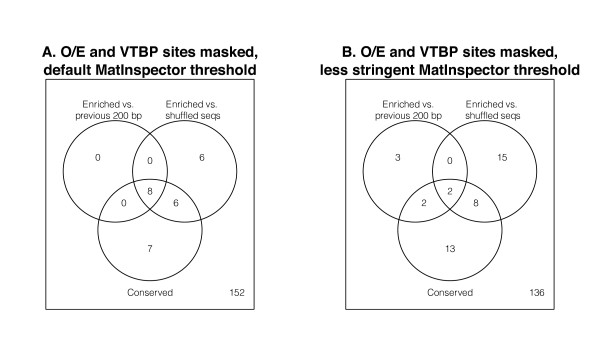
**Overlap between three tests for motif importance**. The Venn diagrams depict the number of matrix families that are significant in each of our three statistical tests after Bonferroni correction, and after masking O/E sites and TATA boxes. Panel A shows results of MatInspector scans using default parameters, and panel B shows results using less stringent MatInspector predictions.

Both tests initially highlighted some potentially interesting candidate transcription factors as being statistically significantly enriched even after a conservative Bonferroni correction for multiple testing (Figures 2A, 2B, 3, Table 1). One example is the V$IKRS matrix family representing binding sites for the Ikaros family (Table 1); these sites have been previously noted in some OR promoters [52]. However, upon closer examination, we find that the binding matrices for Ikaros are rather similar to those for O/E. Although it is possible that additional factors like Ikaros do bind O/E sites, perhaps even in a competitive manner with O/E factors, it seems likely that the apparent enrichment of Ikaros binding sites is merely a consequence of the presence of many true O/E motifs and the sequence similarity between these motifs. In order to search for matrices that are enriched independently of O/E and TATA sites, we masked those predicted motifs from the promoter sequences and repeated our analyses, applying similar masks to the control sets used for our statistical tests in order to ensure that the same amount of sequence was scanned in real and control sets (see Methods).

After masking O/E sites and TATA boxes, 20 matrix families appear significantly enriched by one or both of the tests we describe above, even after applying a conservative Bonferroni correction (Figures 2C, 2D and 3, Table 1, Additional Files 5 and 6). None is enriched to as high a level as O/E sites, but these 20 matrix families are nonetheless found in OR promoters significantly more frequently than expected. We would not necessarily expect more dramatic levels of enrichment than those observed, given that many matrix families are quite degenerate and yield high background levels of false-positive predictions as well as true positives. Below we discuss the candidate transcription factors revealed by our enrichment analysis, but first we describe parallel analyses of evolutionary conservation in the OR promoters.

We also performed all the tests described above on larger promoter regions of 500 bp (for the binomial test, our "control" regions comprised the -500 to -1000 region). We obtained very similar results as we did for the 200-bp promoter regions (Additional Files 7, 8 and 9; e.g. compare Additional File 7 with Table 1).

### Some transcription factor binding sites show orthologous conservation

Functionally important DNA sequences are often evolutionarily conserved, and therefore analysis of aligned orthologous sequences can help identify such regions [42,43]. OR promoters are no exception: sequences near OR TSSs show increased conservation compared to their surroundings (Figure 1A). We sought to dissect the OR promoter regions further to determine whether binding sites for any specific transcription factors accounted for this increased conservation.

We did so by examining conservation scores at the base-pair level to determine whether predicted binding sites for each transcription factor are more conserved than surrounding sequences. This analysis would be difficult for small numbers of promoter sequences, but the size of our promoter dataset gives us the statistical power to perform such analyses. For each of the OR promoter regions, we obtained multiz/TBA sequence alignments of candidate orthologous sequences from up to 20 placental mammals via the UCSC Genome Browser. We then used the SCONE algorithm [45] to estimate the strength of evolutionary conservation at each position of the multiple-sequence alignment. For each PWM family, we examined whether or not conservation within predicted binding sites is higher than that outside the sites (but still within the 200-bp promoter region) (see Methods). We find that O/E sites (predicted using MatInspector's less stringent parameters) show statistically significantly higher conservation than the remaining bases (Table 1, Bonferroni-corrected p < 10^-63 ^considering the whole site, or p < 10^-129 ^considering just the core nucleotides that are most important for binding; one-tailed Wilcoxon tests).

Applying the same statistical test to each of the other matrix families in MatBase, we again initially see significant results for Ikaros and other matrices that share similarity with O/E sites. After masking out O/E sites and VTBP sites and re-applying the statistical tests, we find that 21 matrix families have significantly higher conservation scores (after Bonferroni correction) in cores and/or full sites than surrounding nucleotides, considering MatInspector's default predictions (Table 1, Additional Files 5 and 6). Again, analyzing 500-bp promoter regions instead of 200-bp regions yields very similar set of significant factors.

Although predicted TATA boxes show a clear peak of enrichment close to the TSS, they are not more conserved than surrounding nucleotides. This finding might be due to the short, degenerate nature of this motif (such short sites are easy to recreate at nearby sites during evolution) and/or might reveal some limitations of this test: if the collection of predicted sites being tested contains a large number of false-positive sites (where no conservation would be expected) as well as truly functional sites, the higher conservation scores of real sites would be diluted out by the lower scores of the false-positive sites. The statistical test also has lower power on shorter sites like the TATA box (fewer scores to test) than on longer sites, and conservation will be less impressive for factors that can bind a variety of related sequences (i.e., have degenerate PWMs) than those that require a more exact match.

**Figure 4:**
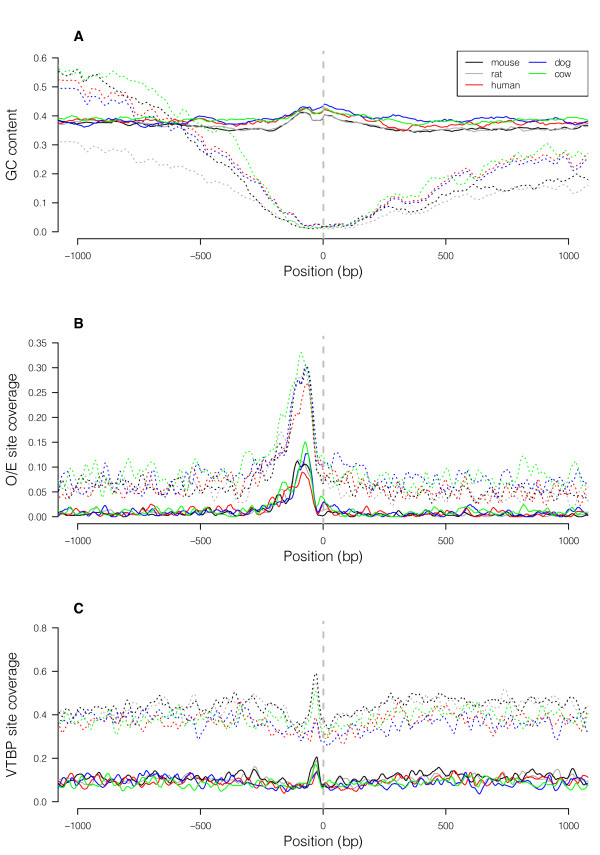
**O/E sites and TATA-boxes are enriched near rat, human, dog and cow OR TSSs**. In all panels, the black lines represent data for the 314 mouse OR promoters (i.e., the same data as shown in Figure 1). Colored lines represent putative orthologous promoter regions (determined using UCSC's liftOver utility) from rat (gray), human (red), dog (dark blue) and cow (green). In panels B and C, solid lines represent matrix matches exceeding MatInspector's default score threshold ("opt"), and dotted lines represent matches found using a less stringent score threshold ("opt-0.1"). Panel A shows that orthologous promoters from all four placental mammals examined exhibit the same reduction in repeat content and characteristic fluctuation in GC content near the predicted TSS. Panel B shows that O/E sites are enriched upstream of orthologous promoters in placental mammals, and panel C shows that TATA-boxes are enriched upstream of orthologous TSSs. As in Figure 1, coverage is calculated as the proportion of promoter sequences containing a predicted O/E (or VTBP) binding site at each base-pair, averaged over 20-bp windows, sliding along promoters 1 bp at a time.

Therefore we used another method to show that OR promoters in other species also appear enriched for TATA boxes. We used cross-species whole-genome alignments to obtain datasets of candidate orthologous OR promoter regions from other mammalian species via UCSC's liftOver utility, which translates coordinates in one genome assembly (in our case the mouse assembly) to orthologous coordinates in other assemblies (see Methods for details, and for an explanation of why we used liftOver rather than the multiple sequence alignments used above). We examined various characteristics of these "lifted Over" promoter regions in several other species and see, in each species examined, similar characteristic patterns of repeat content dips and GC content fluctuations near the predicted TSS as seen for the mouse OR promoters, indicating that at least a majority of the lifted-over promoters are likely to be functional promoters (Figure 4). Upon running MatInspector on the orthologous promoter sets, we also observe enrichment of predicted O/E binding sites and TATA boxes. This analysis demonstrates that TATA boxes are frequently found close to orthologous TSSs, even though they might not conserved in exactly the same location in other species.

### All three statistical tests show the importance of O/E and homeodomain sites

As described above, O/E sites are statistically significantly enriched in OR promoters compared to two sets of background sequences and show statistically significant evolutionary conservation compared to other nucleotides in the OR promoter. After masking O/E sites and TATA boxes, fifteen PWM families show significant results in all three tests when default MatInspector parameters are used. Impressively, eight PWM families are significant in all three tests even after applying the conservative Bonferroni correction for multiple testing (Figure 3, Table 1). All eight of those PWM families represent homeobox transcription factors (Table 1); their enrichment and conservation likely reflect the importance of binding sites for homeodomain proteins Lhx2 and Emx2 (represented by matrices in the V$LHXF and V$HBOX families, respectively) in OR promoters [28-30]. Many of the other homeobox matrices showing significant enrichment have similar consensus sequences to the matrices for Lhx2 and Emx2. As discussed above for Ikaros and the O/E factors, bioinformatic analyses cannot distinguish whether homeobox factors in addition to Lhx2 and Emx2 are involved in OR transcriptional activation, or whether the observed enrichment of additional homeobox matrices is simply due to the high similarity between matrices.

We were curious about the non-homeodomain factors shown by our analyses to be both enriched and evolutionarily conserved. In order to determine which individual factors might be important, we extended our analysis in two ways: (a) we examined enrichment/conservation for individual position weight matrices (Additional Files 10, 11, 12 and 13) rather than for matrix families (Additional Files 5, 6, 7 and 8), and (b) we examined genome-wide expression datasets to determine which of the enriched/conserved factors are expressed in the olfactory epithelium. In more detail, we performed similar analyses to those described above, but using MatInspector's option to report results for individual position weight matrices. As expected, for each family that was significant in our original analyses (Table 1), one or more individual matrices also show significant enrichment and/or conservation. In many cases, a majority of matrices in the family show significant results in one or more tests; this is expected given the similarity of the matrices. Where possible, we determined gene symbols for the corresponding transcription factor for any individual matrix showing significant enrichment and conservation. We then used those gene symbols to look up expression levels according to RNA-seq data generated from mature olfactory neurons and a mix of their progenitors and immature neurons [41], considering factors expressed at high levels in either cell type to be of preliminary interest.

Lhx2, Emx2 and the four O/E factors all stand out clearly when we combine expression data with our enrichment and conservation analyses (Additional Files 10, 11, 12 and 13). In addition to these known factors, the most tantalizing candidate is Nhlh1 (nescient helix loop helix 1, also known as Hen1, Nscl-1 and Tal2). Nhlh1 is represented by a matrix in the V$HAND family that shows modest yet statistically significant enrichment and conservation (Table 1). One of the two individual matrices for Nhlh1 (V$HEN1.01) also shows significant enrichment and conservation when tested alone (Additional File 11). RNA-seq data show that *Nhlh1 *is expressed at high levels in the immature neuron/progenitor cell population (a per gene sum of ~310 reads/exon/kb gene sequence/million reads generated, or fpkm), but only very modestly in mature olfactory neurons (~2 fpkm). We also investigated publically available microarray expression data using the BioGPS interface [53,54]; these data indicate even higher levels of *Nhlh1 *in the vomeronasal organ (VNO) than in the main olfactory epithelium (MOE) and show that *Nhlh1 *is expressed at higher levels in MOE and VNO than in a wide range of other tissues surveyed. Previous studies have implicated *Nhlh1 *in the genesis of GnRH-1 neurons [55], which are born in the olfactory placode and migrate to the brain. Extensive further experimental studies would be needed to determine whether *Nhlh1 *has a role in OR regulation as our bioinformatic tests could suggest, or whether the expression data simply reflect the presence of GnRH-1 precursors among the cells assayed.

### *Ab initio *motif discovery algorithms find O/E and homeodomain-like motifs, but no other motifs

As a complementary approach, we performed *ab initio *motif detection on the OR promoter sequences. Many algorithms exist for this difficult task, and none achieves it perfectly. Tompa *et al. *[56] discuss the challenges of motif detection and compare the performance of various algorithms. No program tested found all of the motif instances planted in the sequences (i.e., all methods had poor site-level accuracy); motif-level accuracy is likely better but was not reported. No algorithm was a clear winner, and those tested appear to have different and sometimes complementary strengths and weaknesses. We decided to use three of the algorithms, MEME [57], Weeder [58] and MotifSampler [59] to search for novel motifs in OR promoters (see Methods). Although we have not tested every possible algorithm on our sequences, the use of three different tools should provide a reasonable chance of detection. However, short, degenerate and/or relatively rare motifs are unlikely to be detectable by any *ab initio *motif detection algorithm, so a truly comprehensive analysis may never be possible.

On the 200-bp promoter sequences, Weeder identifies several O/E-like motifs and several homeodomain-like motifs. MEME identifies a very common motif similar to O/E binding sites, as well as some other marginally significant motifs, including some with weaker similarity to the O/E matrices (data not shown); however, MEME does not report homeodomain-like motif enrichment. MotifSampler yields a similar set of enriched O/E-like motifs but no homeodomain motifs.

On the 500-bp promoter sequences, Weeder identifies only homeodomain-like motifs but not the striking O/E-like motif, whereas MEME and MotifSampler find an enriched O/E-like motif but no enrichment of homeodomain-like sequences.

We repeated the motif searches after masking O/E and VTBP sites as we did for our enrichment analyses above (see Methods); Weeder continues to find homeodomain-like motifs; MotifSampler now identifies homeodomain-like motifs, showing that thorough masking of the most common motif can assist in the identification of secondary motifs. However, MEME reports no remaining statistically significant motifs.

We investigated why MEME and MotifSampler were less effective than Weeder at identifying homeodomain-like motifs on the unmasked promoters. We explored various parameter choices for MEME and MotifSampler and found that the choice of "background model" is influential. When we use a background model derived from 1-kb promoter regions from all known TSSs in the mouse genome (GC content 50.1%; Methods), MEME recovers homeodomain-like motifs (not shown) in addition to O/E-like motifs. When we use a background model supplied by the authors of MotifSampler that was generated from mouse intergenic sequences, we find homeodomain but not O/E-like motifs. However, using our first choice of what seems like a statistically more appropriate choice of background model given the unusual GC content of OR promoters (a model derived from the 4-kb regions surrounding OR TSSs), we found no homeodomain-like motifs. In other words, AT-rich motifs are statistically not very surprising in AT-rich sequences, but are surprising in sequences of genome-wide average GC content. We note that Weeder uses a background model derived from all 1-kb promoter regions (Weeder documentation, version 1.2, 2005). This discrepancy highlights the difficulty of choosing appropriate parameters for motif identification algorithms.

We also used a smaller dataset consisting of evolutionarily conserved promoter subregions as input to MEME, because *ab initio *motif-identification algorithms like MEME should do better on input datasets that are more enriched in true functional elements. We used PhastCons elements [44] present in our OR promoter dataset to define such conserved subregions. The PhastCons program uses a Hidden Markov Model to classify regions of multiple sequence alignments into two states: conserved or neutrally evolving. Considering the 200 bp before the TSS, 68 of the 314 non-redundant OR promoters contain a total of 94 placental mammal PhastCons elements, comprising 3.9% of the basepairs of the promoter regions tested. We eliminated three very short conserved elements (< 8 bp, the minimum size required by MEME), and used the remaining 91 sequences (total length ~2.4 kbp) as input to the MEME algorithm. The only statistically significant motif found is again similar to O/E motifs. MEME reports 74 instances of this motif in 53 of the subsequences, totaling 592 bp, or an impressive 25% of the basepairs of the PhastCons elements used as input.

We also performed MEME analysis on various functionally and phylogenetically defined subsets of the OR promoters, reasoning that some subsets of ORs might share sequence motifs that are not common to the entire OR family, and that analysis of these subsets might reveal motifs that would otherwise be diluted out in the larger, more heterogeneous dataset. The functionally defined subsets we tested include ORs expressed in the same zone of the olfactory epithelium as one another, ORs expressed in the septal organ, ORs whose promoter regions have been shown to interact with the H-region, and ORs for which there is evidence of expression in the vomeronasal epithelium. We also selected phylogenetically defined subsets using either the MOR family categorization [3] or HORDE family assignments [60] or only class I ORs. Upon running MEME on these subsets, we find O/E-like motifs in almost all cases, demonstrating that MEME has the power to find enriched motifs even with very small promoter datasets (as few as 6 sequences). However, after masking O/E motifs, we find almost no significant motifs using MEME in any of the sequence subsets, similar to our findings for the larger sequence dataset (data not shown). The very few statistically significant motifs we do find were rather long and very degenerate, and thus difficult to interpret whether they have any biological significance. Similar analyses using Weeder yielded enriched homeodomain-like motifs in many of the subsets searched, but we did not pick out any common novel enriched motifs among those reported by Weeder. The motifs detected by MotifSampler on these smaller datasets were all rather degenerate and thus it is difficult to interpret whether they are biologically significant.

## Discussion

We have performed rigorous analyses of a large dataset of mouse olfactory receptor promoter regions looking for enriched sequence motifs that might be involved in transcriptional control of this gene family. Our approach combines three statistical tests that deal with issues of signal-to-noise levels that are often problematic when searching promoters with collections of known PWMs. These three tests determine whether or not motifs (a) are enriched in candidate promoter regions relative to the immediately preceding region, (b) are enriched in OR promoters compared to shuffled sequences, and (c) are more evolutionarily conserved than neighboring sequences. We found strong statistical evidence that O/E and homeodomain factors are important in OR transcriptional regulation, agreeing with previous experimental results and with a previous, more limited, bioinformatics search [20]. These results demonstrate that our approach is well designed and effective. However, our analysis revealed no convincing novel candidate transcription factors, suggesting that experimental approaches might have already been successful in uncovering all factors common to the majority of the OR family.

Our analysis focused on either the 200-bp or 500-bp regions immediately upstream of the transcription start site; a 200-bp region appears sufficient to direct appropriate transcription of transgene reporter constructs for the very limited number of OR promoters tested [14,16]. However, it is also possible that some ORs might contain functionally important motifs in other regions, perhaps further upstream of the TSS, in the first intron, 5' UTR, ORF, and/or in the 3' UTR. It is intriguing that olfactory tissues express over 100 microRNAs and that some appear to be necessary as precursor cells differentiate into mature olfactory neurons [61]. Although outside the scope of this study, our statistical approach could also be applied to search for enriched microRNA target sites. A search for DNA- or RNA-based signals residing in the ORF region would also be interesting, but would require different analysis methods that account for the fact that ORFs are constrained by the need to encode functional proteins (and for organismal codon bias) and for variation in conservation levels in different regions of the protein.

Our careful approach highlights some potential pitfalls of the bioinformatic analysis of gene regulation, especially in gene families that include recent duplicates. To ensure independence in statistical tests, we realized it was important to include only one representative of any pair of recently duplicated promoter sequences in any analysis, as false-positive matrix matches would be highly correlated among duplicates and could give false signs of enrichment. We also note that it is difficult to distinguish between transcription factors with similar binding sites (e.g. O/E and Ikaros) and that sequential rounds of analysis can be helpful, masking out sites for the top hit before repeating statistical tests.

Despite our efforts to perform a thorough computational analysis, a bioinformatic approach naturally has many limitations. One of the main issues is that PWMs defining TF binding sites are usually short and quite degenerate, meaning that the matrix will match at many "false positive" locations in addition to true binding sites. Furthermore, PWMs for different TFs have different levels of signal-to-noise, meaning that no single score threshold or filtering strategy is appropriate for all PWMs. Very degenerate binding sites are particularly difficult to detect as enriched: comparison between our observations for O/E and TATA boxes demonstrates this issue clearly. Atypical motifs (e.g., dyads with variable spacers) are also difficult to identify. *In vivo*, local chromatin structure, epigenetic modifications, DNA accessibility, indirect regulation via protein-protein interactions, and perhaps even sub-nuclear localization are likely to be crucial in allowing a transcription factor to act only at the "correct" sites, even if it would be able to bind most predicted sites on isolated DNA *in vitro*.

We note that our use of the Bonferroni correction and the requirement that a factor be recognized by all three tests is a conservative approach - additional PWMs that do not meet formal significance criteria might also be truly enriched and functionally important. Indeed, some motifs (as we have seen for TATA-boxes) might show enrichment in orthologous promoter sets when analyzed independently but fail to show higher conservation scores in multiple sequence alignments because the exact location of the binding site can shift during evolution [62]. Our three statistical tests used a database of transcription factors whose binding sites are known; such databases are almost certainly incomplete. However, our complementary analysis using the *ab initio *motif-identification algorithm MEME revealed a very similar set of enriched motifs as we found using the MatBase database as a starting point. This observation argues that totally novel factors are unlikely to regulate OR promoters unless they recognize very degenerate sequences, which no current bioinformatic approach can detect.

Finally, some members of the OR gene family are expressed outside of the olfactory epithelium as well as in the nose, for example in mouse muscle cells or human testis [63,64], leaving open the interesting possibility that some ORs have been co-opted during evolution to perform new functions. Careful bioinformatic and experimental analysis of promoters of ORs expressed in other tissues could reveal recently arisen transcription factor binding sites that allow novel regulatory control in non-olfactory tissues.

## Conclusions

Our analyses confirm the functional relevance of O/E and homeodomain binding sites across the OR family and suggest that there are no other well-conserved sequence motifs that are important for a majority of the OR family. The mystery of OR transcriptional control remains and will need to be addressed by experimental approaches, perhaps in combination with sequence analyses similar to those we describe here. The recent finding that epigenetic marks may be important in OR regulation [41] opens a promising new line of inquiry.

## Methods

### General bioinformatics resources

Our analyses were performed using custom scripts written in R [65] and PERL, utilizing several Bioconductor packages [66] (principally IRanges) and BioPerl modules [67]. We obtained several genome-wide datasets (including the CpG-island and PhastCons element tracks) via the UCSC Genome Bioinformatics site [68,69] and the Table Browser tool [70]. We also utilized the RepeatMasker program [71] to recognize and mask interspersed repeat sequences from OR promoter regions.

Relative expression levels were derived from RNA-seq data as previously described and provided as a supplement by Magklara *et al. *[41].

### Mouse OR gene dataset

We determined the locations of the 1151 intact OR genes and 287 OR pseudogenes in the most recent version of the mouse genome assembly (July 2007/NCBI37/mm9) using a previously described method [2]. Coordinates of those 1438 OR gene family members are provided in Additional File 1. We determined officially approved gene names (where available) by comparing genomic coordinates of our set with genomic coordinates of existing named ORs according to MGD [72]. Due to changes in genome assemblies, not all ORs in the current assembly have an official gene name. Many OR publications use alternative gene names; these names are included in Additional File 1 when their corresponding sequences match a sequence in our set with 100% nucleotide identity over their full length. Polymorphisms and/or sequence errors might mean that some previously named ORs do not appear in this table.

We defined family and subfamily membership for each mouse OR according to two classification systems: HORDE, determined using human olfactory receptor sequences [4] and MOR, determined using mouse olfactory receptor sequences [3]. For MOR family assignments, we performed a blastn search of each mouse OR we found in the most recent mouse genome assembly against nucleotide sequences of a set of mouse ORs named according to a different classification system [73]. We assigned each mouse OR membership in the same MOR family as its best match in that blastn search. For HORDE family/subfamily assignments, the reference human sequence set is more diverged from our mouse query sequences than is the MOR reference set, making a blastp search more appropriate. Therefore, we used translated mouse OR ORF sequences to search a local file containing predicted human OR protein sequences from the HORDE database (version 41) [4,74]. If the closest-matching human gene in that blastp search showed at least 40% amino acid similarity with the mouse OR query, we gave that mouse gene the same HORDE family assignment as its human target; if the match exceeded 60% similarity, we also assigned the same subfamily as the human target.

### Defining TSS locations

We previously reported end-sequences of 1264 OR cDNAs from adult and embryonic olfactory epithelial cDNA libraries [13] among a larger set of 1738 sequences [Genbank: CB172832 - CB174569]. We excluded some cDNAs that showed unusual splice patterns [13] and a small number of sequences derived from the 3' ends of the clones. We also analyzed 434 5'-RACE sequences reported by Michaloski *et al. *[20] [Genbank: DR065530 - DR065963].

In order to assign each EST or RACE sequence to the corresponding OR gene, we first defined a genomic "domain" for each OR, whereby any cDNA mapping to that domain on the correct strand would be assigned as being transcribed from the corresponding gene. We defined the upstream edge of the domain as being 1 kb closer than the nearest edge of the next OR ORF upstream (or 100 kb upstream of the ORF's start if no other OR was found within that distance); we defined the downstream edge of the domain as being 1 kb downstream of the end of the gene's ORF.

We used cDNA or RACE product sequence accession numbers as queries in UCSC's Table Browser to look up their map positions in the mouse genome assembly (July 2007). We then used Bioconductor's IRanges package to compare those cDNA positions to the set of OR gene domains as defined above. cDNAs whose position overlapped more than one OR domain were excluded from further analysis. For each gene domain, we assigned base-pair positions present in any cDNA/RACE sequence and/or in the ORF of the gene domain as part of the transcript. Because UCSC's cDNA-genome mapping algorithm reports separate alignment blocks whenever an indel occurs, we merged any transcribed segments that were separated by < 10 bp, as these gaps are very unlikely to represent true introns. After this merge, we removed from further analysis 49 OR genes for which only a single transcribed exon appeared to be present, as these might represent cDNAs that are only partial transcripts or genomic contaminants of the cDNA library. We also removed 11 apparent transcribed pseudogenes (these transcripts might truly originate from a pseudogene, or the OR might be intact in the mouse in which transcripts were observed, with a single nucleotide polymorphism or sequence error causing it to appear as a pseudogene in the genome assembly). We removed 2 additional ORs for which the cDNA/RACE sequences implied an unusual gene structure (an intron downstream of the ORF), leaving 432 intact ORs for which we assigned the furthest upstream base of the transcript as the TSS.

In order to ensure that our final dataset did not contain any close duplicates, we grouped the 1151 intact ORs by sequence similarity. We determined pairwise synonymous nucleotide divergence of the 1151 intact OR coding regions using an in-frame nucleotide alignment as input to PAML's codeml algorithm (version 4.3) [75], run in pairwise mode, and identified any pairs of ORs showing divergence of < 0.3 substitutions per synonymous site as "similar". We also used blastn to cross-compare 2 kb of sequence around each of the 432 TSSs (removing any ORF sequence, if the TSS was closer than 1 kb to the ORF start). We assigned as "similar" any pair of 2-kb sequences that blastn was able to align for at least 200 bp with at least 70% nucleotide identity. We used those two lists of "similar" OR pairs as input for a Perl script that performs single linkage clustering and then arbitrarily chose only one OR from each group for further analysis. The resulting dataset of 312 ORs should therefore contain no pairs of recently duplicated ORs.

### MatInspector analysis

In order to search for candidate binding sites for transcription factors with known sequence specificity, we purchased a license to use the MatBase database and MatInspector search algorithm [49] (Genomatix Software GmbH, Munich, Germany). We used a local installation of the Matrix Family Library (Version 8.02, January 2010) and software (Version 8.20 Professional, February 2010) and searched genomic sequences near OR TSSs with all PWMs in the "vertebrate" and "general core promoter elements" categories of the library. MatBase groups PWMs that represent the same or functionally similar transcription factors into families: the version of MatBase that we searched contains 727 vertebrate PWMs in 170 families and 16 general core promoter element PWMs grouped into 10 matrix families. The total number of matrix families considered in our search was 177 (we ignored three matrix families in the general category that on closer inspection were found to be fly-, plant- or yeast-specific). Unsurprisingly, similar matrices often recognize overlapping or identical regions of a query promoter sequence; MatInspector helps reduce an overwhelming number of predicted sites by reporting only the highest-scoring matrix match per family in any overlapping region of the query sequence. We therefore performed most of our statistical analyses at the level of MatBase families rather than on individual PWMs.

We ran MatInspector twice with different parameters; once using the default score threshold (called the "optimized" threshold, referred to in the manuscript as "opt"), and again using a threshold 0.1 units lower than the optimized threshold ("opt-0.1"), which results in about ten times as many matches as does "opt". We used the OUTFILES = 2 option to obtain tab-delimited text output, which we then parsed and analyzed in R. Many transcription factor binding sites are at least partially palindromic, so that MatInspector often predicts overlapping sites for the same matrix on opposite strands.

### Enrichment statistics

Transcription factor binding sites are represented by short and degenerate position weight matrices (PWMs). This degeneracy means that when genomic sequence is searched with a PWM, there is often a non-trivial level of false-positive site predictions. We wanted to determine whether the number of predicted sites in the 200-bp regions nearest our set of predicted TSSs was more than would be expected in "background" non-promoter sequences. We also performed all the tests described below on 500-bp regions upstream of the TSS: for simplicity, below we describe analysis of only the 200-bp regions. As described in the main text, we performed two statistical tests to address this question, using two different sets of background sequences. In the first test, we compared the number of predicted sites in the 200 bp nearest to the predicted TSS with the number predicted in the preceding 200-bp region. It is possible that some sites in that previous 200-bp region do indeed represent real TF binding sites, reducing the ability of our test to detect truly functional sequence motifs in the TSS-proximal region, but given the unusual GC-content of our promoters, we decided that it was best to use neighboring regions as controls rather than anything yet farther from the predicted TSSs. We used a one-tailed binomial test to assess whether the number of sites in our regions of interest was statistically significantly greater than that in the background region, with a null hypothesis that transcription factor binding sites are equally likely to be found in each of the 200-bp regions. Because we performed each test on 177 PWM families, we conservatively required a p-value of < 0.05/177 (< 0.000282) in order to declare statistical significance (the Bonferroni correction).

In our second test, we randomized the DNA sequence of each 200-bp promoter region, using the shuffle program from the SQUID package (version 1.9g, S. Eddy) [76], supplying a random seed and maintaining mononucleotide and dinucleotide composition by specifying the "-d" flag. We repeated the shuffling process 10,000 times, supplying a different random seed each time, in order to obtain 10,000 datasets of "negative control" sequences with the same nucleotide composition as our real 200-bp promoter regions. We ran MatInspector on these shuffled datasets in the same way as we had with the real sets and tabulated for each dataset the number of predicted sites found for each matrix family. We then counted how many of the 10,000 shuffled sets contained a number of matrix matches that exceeded that found in the real promoters, taking that proportion as a "shuffled P-value", again using a conservative Bonferroni-corrected threshold of p < 0.000282 to declare statistical significance. We also calculated the mean number of matrix matches found across the 10,000 sets; comparison with the number found in the real dataset gives an estimate of enrichment level. An explanatory example is given in the main text.

We investigated the use of an alternative type of negative control sequence: GC-matched promoters from non-OR genes, as one would not expect many of them to share the same enriched motifs as OR promoters. However, it proved impossible to assemble a truly appropriate non-OR promoter control set. First, OR promoters have such extremely low GC content that we struggled to obtain a matched set; only ~280 promoters in the mouse genome had similarly low GC content. Those GC-matched non-OR promoters still had much higher conservation scores than our OR-promoter set (data not shown) - it was thus not possible to match promoters on GC content and conservation levels. This difference in conservation levels is difficult to interpret given the fact that the OR family has experienced many recent duplications and deletions disrupting orthology, and given that OR coding regions appear to be evolving under much lower selective constraints in some species (e.g. primates) than others (e.g. rodents), so that it seems likely their promoter regions are also under lower selective constraint. The sequences we analyzed that immediately precede the OR promoter sequences are only very slightly different in GC content and conservation level to the promoter dataset (Figure 1), and are better matched for these parameters than any non-OR promoter dataset we could have assembled.

### Analysis of conservation scores

We used UCSC's Table Browser to obtain multiple-species alignments of the 200-bp promoter regions. These are portions of whole-genome alignments that were generated by a sophisticated computational pipeline based on the multiz and TBA programs [77]. One caveat of multiz/TBA alignments is that they may contain a minority of spuriously aligned regions [78]. In a study assessing the accuracy of various whole-genome alignment methods [78], another program, PECAN [79] was found to produce more accurate alignments. However, we found that in the olfactory receptor promoter regions, PECAN aligned very little sequence at all (data not shown), perhaps due to a very conservative approach in regions that have experienced post-speciation duplication. We reasoned that even if the multiz/TBA alignments contain a minority of misaligned regions, and might have aligned a subset of sequences from other species to more than one mouse location, they would likely still provide some power in looking for conserved subregions of OR promoters. We chose to use aligned sequences from only placental mammals, because our examination of OR region alignments in the UCSC Genome Browser revealed that placental mammal alignments are usually part of longer chained alignments, whereas apparently aligned sequences from other species are often fragmentary and confined to interspersed repeat elements.

Several algorithms exist that estimate the evolutionary selective pressure acting on each nucleotide of a set of aligned sequences, given a phylogenetic tree defining the evolutionary relationships between the sequences. We chose to use the SCONE algorithm [45], subtracting SCONE's "Pval" score (represents the probability that a site is evolving neutrally) from 1 to obtain a score that is higher for more conserved residues. We ran SCONE on each of the multiple-sequence alignments, using a species tree obtained from the UCSC Genome Bioinformatics site [80] that we manually pruned so that it contained only placental mammals. We parsed SCONE output using Perl and R/Bioconductor. For each family of transcription factor matrices in MatBase, we used a one-tailed Wilcoxon test to determine whether or not SCONE scores of alignment positions inside predicted binding sites are higher than SCONE scores outside of sites. We performed two versions of the test: (a) we examined all bases of the predicted TF binding sites, and (b) we examined just the four most essential "core" positions of the binding region, as defined in MatBase. One might expect higher conservation in the core residues and thus a greater distinction between site and non-site scores; however, many fewer alignment positions exist in the cores than the whole sites, and in some cases the increased statistical power gained by considering the whole site meant that we obtained a significant result using the whole sites but not the cores. We therefore recorded as significant any matrix family that appeared interesting in either the whole site or the core test (although both tests showed significance in most cases), after applying the same conservative Bonferroni correction described above.

### OR promoters in other species

In order to examine OR promoters in other species, we used UCSC's liftOver utility to obtain orthologous TSS coordinates in the rat, human, dog and cow genomes. For a given base-pair range in a reference genome assembly, liftOver provides the coordinates of the aligned region from another species, using the same whole-genome alignments we discuss above. We considered an alternative approach of using the orthologous sequences present in the multiple sequence alignments discussed above, but in many cases the alignments contained < 1 kb of orthologous sequence concentrated around the TSS, and in some cases aligned sequences comprised several non-contiguous pieces of the orthologous genome. We wished to examine a more extended, continuous stretch of sequence centered around the orthologous promoter, rather than only those regions that were found to align using whole-genome alignment procedures. As input to liftOver, we used 21-bp regions centered around the predicted TSSs (smaller regions often failed to "liftOver" due to frequent small insertions/deletions in the alignments). After obtaining orthologous coordinates for those 21-bp regions, we took the center position as the predicted orthologous TSS, and extended 2 kb each side of that TSS to create 4-kb regions. The resulting dataset of 4-kb regions contained some redundancy (e.g., in some cases, several mouse promoters pointed to the same or overlapping orthologous human promoters, indicating a likely duplication in the mouse genome since divergence from human, or a deletion of one paralog in human). We removed such redundancy by merging overlapping 4-kb regions, and taking the most upstream 4 kb of each merged region (again, effectively using the most upstream of possible alternate promoters in our analysis). Almost all of the predicted orthologous promoter regions are near OR genes in the corresponding genomes.

### Conservation of O/E sites predicted at less stringent MatInspector parameters

In order to determine the proportion of O/E sites predicted using MatInspector's less stringent parameters that appear evolutionarily conserved, we performed the following analysis, which utilizes the mammalian species tree and multiple sequence alignments of OR promoter regions described above. We used ungapped versions of each sequence in the multiple sequence alignments as input to MatInspector (again using the less stringent parameters). We used a custom Bioconductor script to convert the position of all predicted O/E sites in ungapped sequences to positions relative to the multiple sequence alignment, and therefore relative to the reference mouse sequence. For each mouse O/E site, we could then determine the list of species that had overlapping O/E site predictions. We used that species list to extract a subtree from the full species tree and determined the length of that subtree: subtree length thus provides a measure of how evolutionarily conserved each site is. We then filtered mouse O/E sites according to various minimum tree length thresholds (Additional File 3, panel A). Full details on tree construction and units of branch length are given by UCSC [81]. To give the reader some sense of how tree length translates to which of the species possess a site, some examples of subtrees and their total branch lengths are given here: mouse-rat = 0.161; mouse-human = 0.453; mouse-rat-human = 0.537; mouse-rabbit-guinea pig = 0.723; mouse-chimpanzee-cat = 0.760; mouse-rat-guinea pig-hedgehog-tenrec = 1.24.

Recent work has shown that transcription factor binding sites sometimes show turnover, that is they are found in orthologous promoter sequences but not at exactly orthologous locations [62]. To explore this idea in OR promoters, we repeated the above analysis, but instead of requiring sites to overlap in the multiple species alignment we allowed various amounts of "slide": e.g., if there was a human O/E site at a location that aligned within 50bp of a mouse O/E site, we scored that mouse O/E site as being conserved in human (Additional File 3, panel B). This approach is very successful at selecting O/E sites that are likely to be functional (Additional File 3). In the text, we discuss "conserved" O/E sites: we selected sites conserved with a tree length of at least 0.17 (i.e., conserved in at least one additional mammal, other than rat), allowing 50bp slide (green line, Additional File 3, panel B). For other matrix families, different thresholds/slide amounts appeared more successful, and for some matrix families (e.g. O$VTBP, representing TATA boxes), no combination of slide amount/tree length appeared able to select likely functional sites making it difficult to systematically implement such an evolutionary filtering approach. This topic is discussed at more length by Kheradpour *et al. *[82].

### Masking O/E sites and TATA-boxes from promoter sequences

As explained in the main text, we masked O/E sites and candidate TATA-boxes from the promoter regions and repeated our analyses to determine whether any matrix families show enrichment or conservation independently of O/E and TATA-boxes. For the purpose of masking, we decided to be fairly loose in our definition of O/E sites because any sites left in the dataset could still yield apparent enrichment of O/E-like sequences that is not truly independent of O/E enrichment. We therefore masked out the entire extent of any O/E site predicted by MatInspector at our lower stringency setting ("opt-0.1"). We also masked all predicted TATA boxes ("opt") between position -61 and -10 relative to the TSS. We recognize that this approach is very conservative (masks more sequence than necessary) because there is a relatively high false-positive prediction rate for TATA boxes; we preferred to mask too much sequence than to identify enrichment of transcription factors with TATA-box-like matrices.

As well as masking those regions from the real promoter set, we recorded the positions of the masked sites and also masked the equivalent regions from (a) each of the 10,000 shuffled datasets, (b) the dataset of preceding 200-bp regions, and (c) the lists of conservation scores. We then repeated MatInspector runs and the three statistical analyses using masked sequences and scores. This strategy ensured that an equivalent amount of sequence was being analyzed in the real and control datasets.

### Identification of novel sequence motifs

We used three programs (MEME, Weeder and MotifSampler) to identify novel sequence motifs using various sets of RepeatMasked promoter region sequences as input.

First, we used MEME [57] to search both strands of the input sequence set (parameter: -revcomp) for the ten best motifs (parameter: -nmotifs 10) of size 4-10 bp (parameters: -minw 4 -maxw 10). We also tried searching for motifs of size 4-25 bp: results were very similar. We allowed motifs to occur any number of times in each sequence of the input set (-mod anr), searching for up to N*3 motif instances in the dataset, where N is the number of input sequences (-maxsites N*3). We used the MEME suite's fasta-get-markov program with option -m 2 to construct a second-order Markov model of single nucleotide, dinucleotide and trinucleotide frequencies as the background model of nucleotide frequencies (MEME parameter: -bfile). Input sequences for fasta-get-markov were RepeatMasked 2-kb regions centered around our set of 314 candidate TSSs. We also tested MEME's performance using a different background frequency model derived from all 1-kb promoters in the mouse genome (more GC-rich than our promoter set; 50.1% versus 37.0% GC for the equivalent 1kb region in OR promoters). This promoter set contains 23271 sequences and was obtained (July 16^th^, 2010) from the Genome Bioinformatics site [64].

We ran Weeder [58] locally using the "weederlauncher.out" helper script provided by the authors, which searches for motifs of length 6, 8, 10 and 12bp (allowing 1, 2, 3 or 4 mutations, respectively). By default, Weeder assumes the motif is present one or more times in at least half of the input sequences. We specified the "S" option to search both strands of the input sequences and the "MM" species option so that a background sequence model derived from mouse genomic sequences would be used. It is not possible to specify a custom background model for promoters of unusual GC-content. The program finds the best 10 motifs of each length, and then compares all motifs identified, reporting groups of similar motifs as "redundant" motifs. Weeder gives no p-value or confidence estimate for whether reported motifs are truly enriched.

For MotifSampler [59], we first created a second-order background model from our set of 2kb promoter regions using the CreateBackgroundModel algorithm from MotifSuite [83]. We then ran MotifSampler using this background distribution and the -n 4 parameter (so that the best four motifs would be found). By default, MotifSampler performs its analysis 100 times using different starting points each time. We processed MotifSampler's results using the MotifRanking algorithm which combines similar motifs found in the different "replicate" runs; as the authors advise, we only retained motifs that were found in at least 30/100 of those analyses.

## List of abbreviations used

CAGE: cap analysis of gene expression; EST: expressed sequence tag; LCR: locus control region; OR: olfactory receptor; ORF: open reading frame; PWM: position weight matrix; RACE: rapid amplification of cDNA ends; RNA-seq: massively parallel cDNA sequencing; TF: transcription factor; TSS: transcription start site; UTR: untranslated region;

## Competing interests

The authors declare that they have no competing interests.

## Authors' contributions

JY designed the study, analyzed the data and drafted the manuscript; RL and BT participated in study design and edited the manuscript; RL performed initial data analyses. All authors read and approved the final manuscript.

## Supplementary Material

Additional file 1 **Table listing olfactory receptor genes and pseudogenes in the July 2007 mouse genome assembly**. See Methods for details of how gene names were determined. Click here for file

Additional file 2 **Table listing TSS locations and data sources**. This table shows all TSS locations we collected from a high-throughput RACE study [20], our hybridization-based cDNA screen [13] and various other RACE experiments performed on smaller numbers of ORs [6,14,17,18,52,85-87]. For some ORs, more than experiment defines a TSS (so there are multiple rows for some genes in this table). The "Best TSS?" column indicates whether each defined TSS is the most upstream one for that gene ("Best"), or lies downstream of another TSS for the same gene ("NotBest"). The most upstream TSS for each gene is included in Additional File 1 and was used in our promoter analyses. * Gene names are given where the TSS location was derived from RACE experiments (excluding the high-throughput RACE experiments of Michaloski *et al.*). Click here for file

Additional file 3 **Figure showing that most O/E sites predicted using MatInspector's less stringent parameters are evolutionarily conserved**. The solid gray line shows all mouse O/E sites predicted using MatInspector's less stringent parameters. For reference we include a dotted gray line showing all mouse O/E sites predicted using MatInspector's default parameters. As in Figure 1, coverage is calculated as the proportion of promoter sequences containing a predicted O/E binding site at each base-pair, averaged over 20-bp windows, sliding along promoters 1 bp at a time. In panel A, the colored lines show predicted mouse O/E sites that remain after we apply an evolutionary "filter" to MatInspector predictions on a multiple sequence alignment (see Methods). For example, the red line shows mouse O/E sites that have overlapping O/E sites in a set of mammals with tree length of at least 1.5. Note that the conservation filter removes many of the O/E sites far away from the TSS and selects for likely functional sites near the TSS. In panel B, we explore the effect of allowing various amounts of "slide" (see Methods), where sites in other species are no longer required to overlap but could be various distances away, accounting for evolutionary "turnover" [62]. Click here for file

Additional file 4 **Figure showing enrichment of transcription factor binding sites 200 bp before TSS using less stringent MatInspector parameters**. These plots follow the same layout as those in Figure 2 but use transcription factor site predictions made using MatInspector with less stringent parameters (see Methods). Click here for file

Additional file 5 **Table summarizing statistical tests on matrix families for 200-bp promoter region (default MatInspector parameters)**. Results of our statistical tests for enrichment and conservation for all matrix families after masking O/E sites and TATA boxes using default MatInspector parameters. See legend to Table 1. Click here for file

Additional file 6 **Table summarizing statistical tests on matrix families for 200-bp promoter region (less stringent MatInspector parameters)**. Results of our statistical tests for enrichment and conservation for all matrix families after masking O/E sites and TATA boxes using less stringent MatInspector parameters. See legend to Table 1. Click here for file

Additional file 7 **Summary of statistical tests for 500-bp promoter region**. See legend to Table 1. This table gives equivalent statistics for a larger putative promoter region comprising 500bp before the TSS. Binomial tests here show comparison with the preceding 500-bp region. Click here for file

Additional file 8 **Table summarizing statistical tests on matrix families for 500-bp promoter region (default MatInspector parameters)**. See legend to Additional File 5. Click here for file

Additional file 9 **Table summarizing statistical tests on matrix families for 500-bp promoter region (less stringent MatInspector parameters)**. See legend to Additional File 6. Click here for file

Additional file 10 **Table summarizing statistical tests on individual matrices for 200-bp promoter region (default MatInspector parameters)**. Results of our statistical tests for enrichment and conservation for individual matrices after masking O/E sites and TATA boxes using less stringent MatInspector parameters. See legend to Table 1. In addition, for selected matrices, we obtained gene symbols of the corresponding transcription factor(s) (using MatBase and/or the Mouse Genome Database: [72]), and used gene symbols to query RNA-seq data generated from mature olfactory neurons and a mix of immature neurons and precursors [41]. We provide here the gene symbol and two numbers representing expression levels in OMP+ cells (mature olfactory neurons) and NGN+ cells (immature olfactory neurons and precursors), respectively (see Methods). Click here for file

Additional file 11 **Table summarizing statistical tests on individual matrices for 200-bp promoter region (less stringent MatInspector parameters)**. Results of our statistical tests for enrichment and conservation for individual matrices after masking O/E sites and TATA boxes using less stringent MatInspector parameters. See legend to Table 1 and Additional File 10. Click here for file

Additional file 12 **Table summarizing statistical tests on individual matrices for 500-bp promoter region (default MatInspector parameters)**. See legend to Additional File 10. Click here for file

Additional file 13 **Table summarizing statistical tests on individual matrices for 500-bp promoter region (less stringent MatInspector parameters)**. See legend to Additional File 11. Click here for file
