# Supplementary material for: Rigorous and thorough bioinformatic analyses of olfactory receptor promoters confirm enrichment of O/E and homeodomain binding sites but reveal no new common motifs
Source: BMC Genomics. 2011 Nov 15;12:561. doi: 10.1186/1471-2164-12-561 (PMC3247239; doi:10.1186/1471-2164-12-561)

**Additional Data File 3.**
Distribution of conserved predicted O/E sites

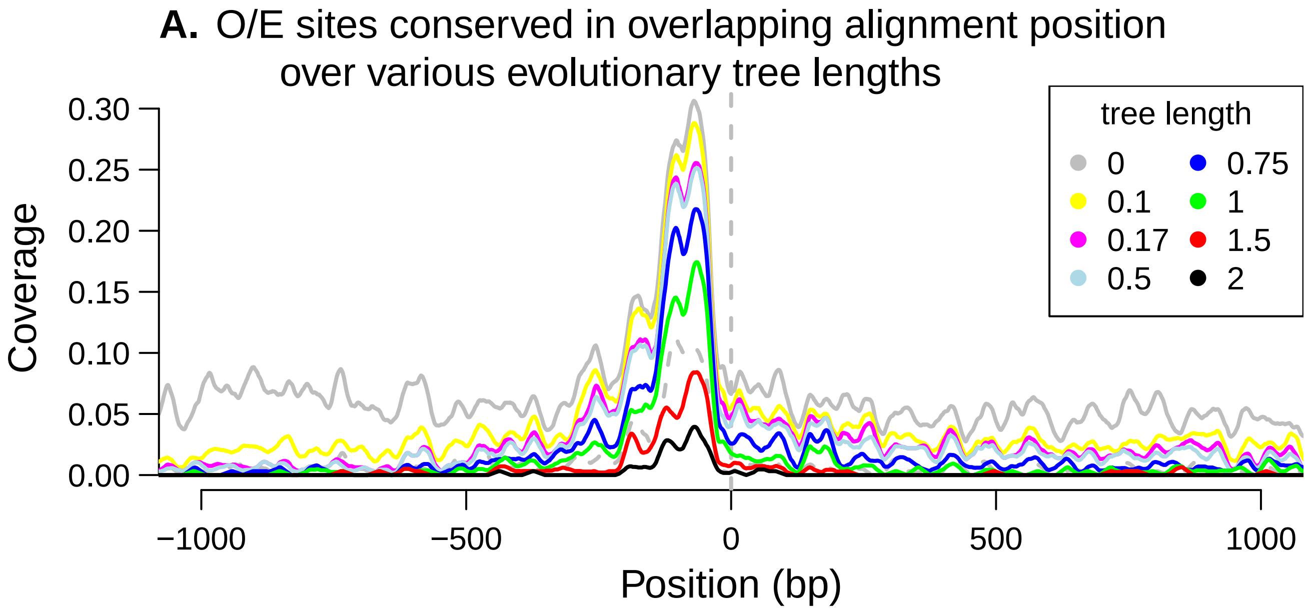


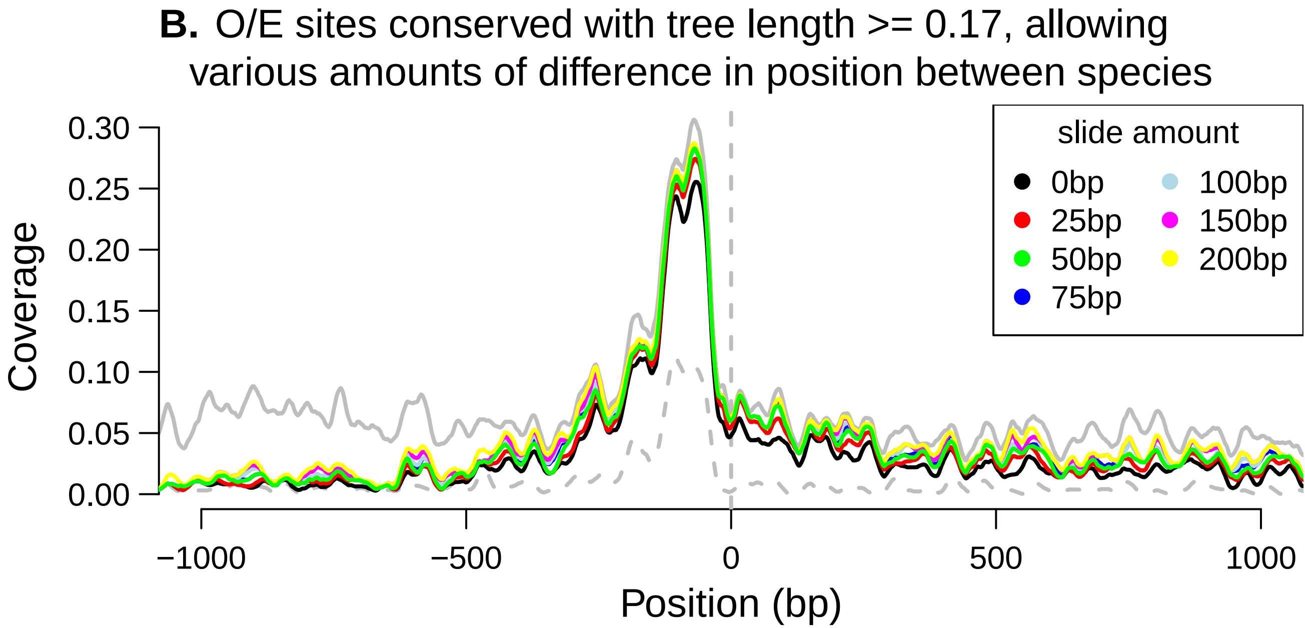

Supplement: Additional file 3 — Figure showing that most O/E sites predicted using MatInspector's less stringent parameters are evolutionarily conserved. The solid gray line shows all mouse O/E sites predicted using MatInspector's less stringent parameters. For reference we include a dotted gray line showing all mouse O/E sites predicted using MatInspector's default parameters. As in Figure 1, coverage is calculated as the proportion of promoter sequences containing a predicted O/E binding site at each base-pair, averaged over 20-bp windows, sliding along promoters 1 bp at a time. In panel A, the colored lines show predicted mouse O/E sites that remain after we apply an evolutionary "filter" to MatInspector predictions on a multiple sequence alignment (see Methods). For example, the red line shows mouse O/E sites that have overlapping O/E sites in a set of mammals with tree length of at least 1.5. Note that the conservation filter removes many of the O/E sites far away from the TSS and selects for likely functional sites near the TSS. In panel B, we explore the effect of allowing various amounts of "slide" (see Methods), where sites in other species are no longer required to overlap but could be various distances away, accounting for evolutionary "turnover" [62]. [file 1471-2164-12-561-S3.PDF]
