# Supplementary figures and images for: Rigorous and thorough bioinformatic analyses of olfactory receptor promoters confirm enrichment of O/E and homeodomain binding sites but reveal no new common motifs
Source: BMC Genomics. 2011 Nov 15;12:561. doi: 10.1186/1471-2164-12-561 (PMC3247239; doi:10.1186/1471-2164-12-561)

**Additional Data File 4. Enrichment of transcription factor binding sites 200 bp before TSS**

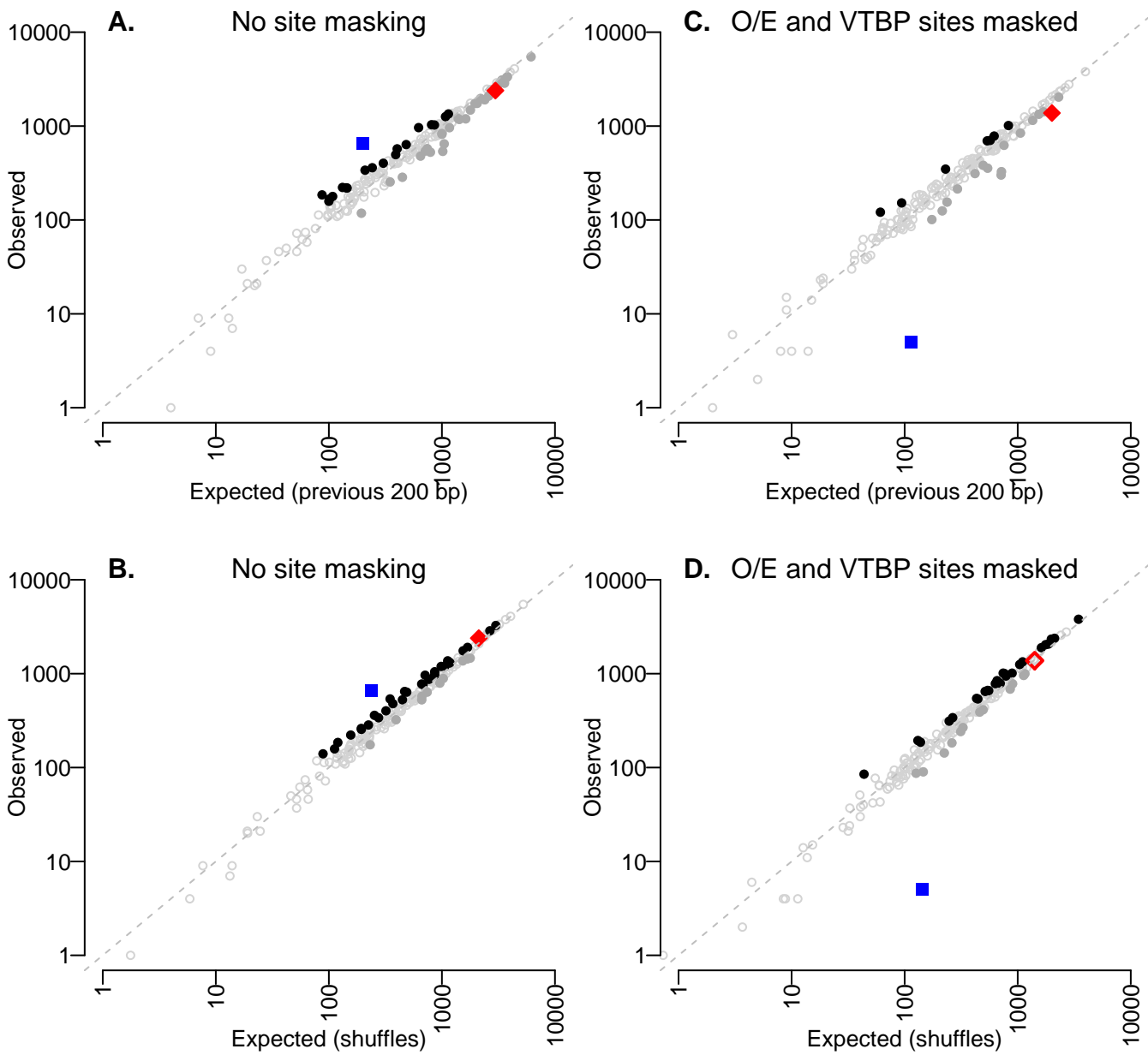

Supplement: Additional file 4 — Figure showing enrichment of transcription factor binding sites 200 bp before TSS using less stringent MatInspector parameters. These plots follow the same layout as those in Figure 2 but use transcription factor site predictions made using MatInspector with less stringent parameters (see Methods). [file 1471-2164-12-561-S4.PDF]
